# Case report: A rare case of very well-differentiated gastric adenocarcinoma of gastric type with a lymphovascular invasion

**DOI:** 10.3389/fonc.2024.1396281

**Published:** 2024-04-25

**Authors:** Jiaqi Chen, Xiujie Cui, Honglei Wu, Chengjun Zhou

**Affiliations:** ^1^ Department of Pathology, The Second Hospital of Shandong University, Jinan, Shandong, China; ^2^ Department of Pathology, The Second Hospital, Cheeloo College of Medicine, Shandong University, Jinan, Shandong, China; ^3^ Department of Gastroenterology, The Second Hospital, Cheeloo College of Medicine, Shandong University, Jinan, Shandong, China

**Keywords:** very well-differentiated gastric adenocarcinoma, microsatellite instability, submucosal infiltration, lymphovascular invasion, case report

## Abstract

**Background:**

Very well-differentiated gastric adenocarcinoma (VWDA) is a rare variant of gastric cancer, for which the diagnostic criteria and clinical behavior are not fully established. We reported a case of an intramucosal VWDA of gastric type with a lymphovascular invasion (LVI).

**Case presentation:**

A 67-year-old female was diagnosed as intramucosal gastric adenocarcinoma after a biopsy at the local hospital 3 weeks ago and then visited our hospital for further treatment. The endoscopic examination in our hospital showed a rough, slightly faded, 30-mm, flat, and elevated lesion on the lesser curvature of the middle gastric body. Histopathologically, the lesion consisted of superficial foveolar-type papillary adenocarcinoma and deep pyloric gland-type tubular adenocarcinoma. The immunostaining results showed that the foveolar-type papillary adenocarcinoma was positive for MUC5AC and had a high index of Ki-67, but the pyloric gland-type tubular adenocarcinoma was positive for MUC6 and had a low index of Ki-67. Both components were negative for MSH2 and MSH6, which suggested the high microsatellite instability phenotype. Moreover, a LVI was detected in the lesion. The pathological diagnosis was VWDA of gastric type.

**Conclusion:**

The case has unique histological and immunophenotypic characteristics, which not only indicates the importance of architectural features in the diagnosis of VWDA but also further proves that the aggressive behavior of VWDA is correlated with tumor histological type and immunophenotype.

## Introduction

Very well-differentiated gastric adenocarcinoma (VWDA) is a rare variant of gastric adenocarcinoma, which accounts for 0.2%–1.9% of all primary gastric cancers (1). It is defined as neoplastic lesions composed of a highly differentiated neoplastic epithelium which mimics the normal gastric mucosa or intestinal metaplastic mucosa with mild nuclear atypia but has the ability to invade the gastric wall (2). In recent years, Japanese scholars (3) have classified the histological types of low-grade well-differentiated gastric adenocarcinoma and clearly pointed out that VWDA (immunophenotype including gastric type, gastrointestinal type, and intestinal type) is an independent tumor type. Due to the low incidence rate and lack of specific characteristics, it is easy for pathologists to misdiagnose VWDA as intestinal metaplasia, especially on biopsy specimens (4). Herein we report a rare case of an intramucosal VWDA of gastric type with lymphovascular invasion (LVI) and hope to improve the understanding of this tumor.

## Case report

A 67-year-old woman experienced nausea and non-projectile vomiting accompanied by upper abdominal discomfort, abdominal distension, fatigue, and poor appetite approximately 20 days ago. The vomit was stomach contents, and the nausea was relieved after vomiting. The patient had no obvious cause of the nausea or vomiting and no history of gastrointestinal bleeding. After undergoing upper gastrointestinal endoscopy and biopsy at the local hospital, the patient was diagnosed with intramucosal gastric adenocarcinoma. Then, she came to our hospital for further diagnosis and treatment. Both the physical examination and the routine laboratory investigations were unremarkable.

**Figure 1:**
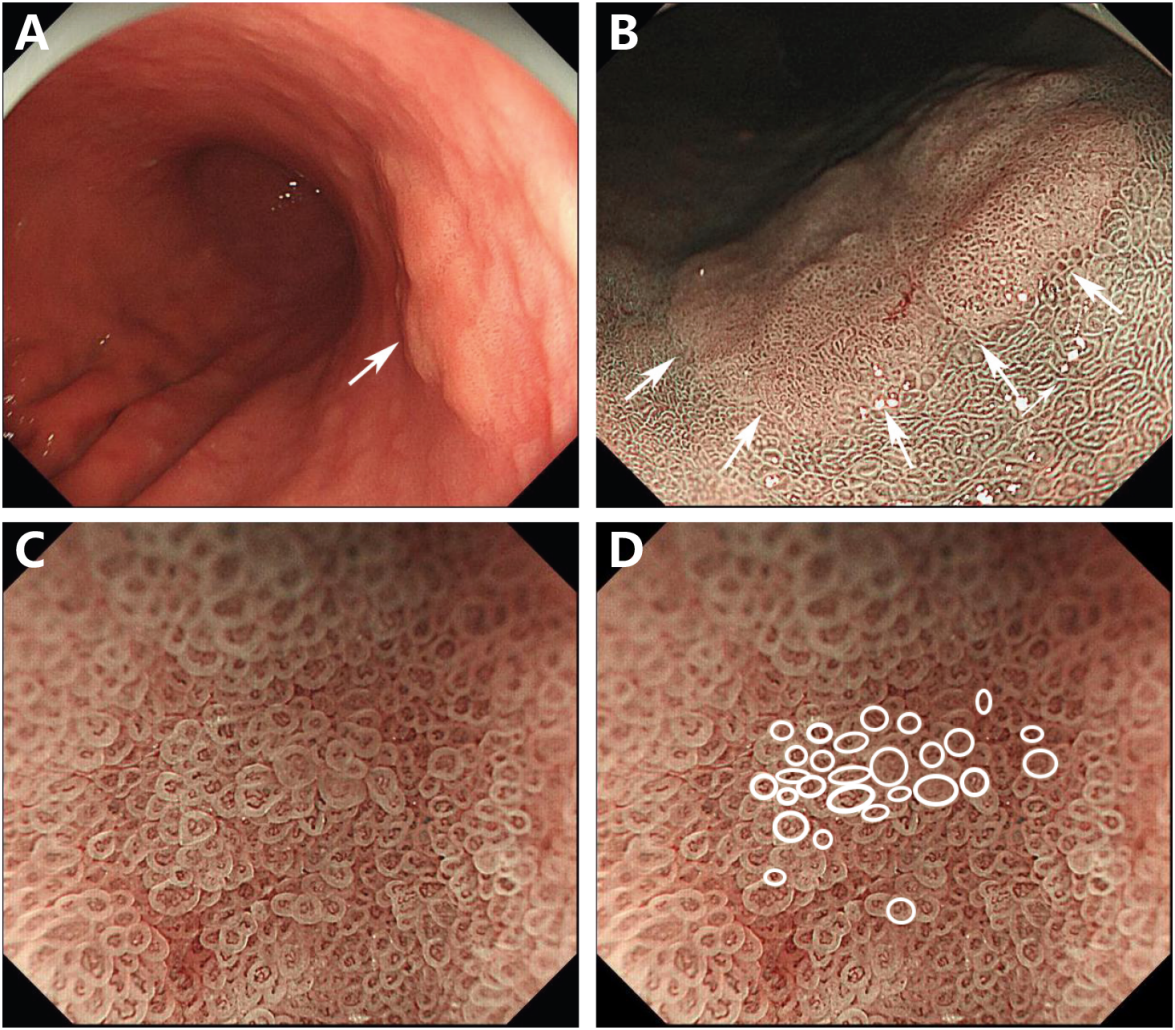
Endoscopic images. Endoscopic examination revealed a 30-mm, flat, elevated lesion on the lesser curvature of the middle gastric body (arrow) **(A)**. NBI showed a defined, slightly faded elevated area within a demarcation line (arrows) **(B)**. M-NBI revealed that the lesion exhibited the vessels within an epithelial circle pattern. A dilated and irregular microvascular pattern was observed in the circular intervening part between crypts lined by circular marginal crypt epithelium **(C)**. The circular marginal crypt epithelium was traced **(D)**.

The endoscopic examination in our hospital showed that there was a slightly faded, rough-surfaced, flattened, elevated lesion on the lesser curvature of the middle gastric body. The background of the lesion mucosa was pale, the mucosal vessels’ visibility increased, and the gastric folds disappeared, which were the specific features of atrophic gastric mucosa (**Figure 1A**). There was no suspected hard-texture area in the lesion, so the depth of the lesion was expected to be intramucosal. The narrow-band imaging (NBI) result showed a well-defined, slightly faded elevated area within a demarcation line (**Figure 1B**), and the size of the lesion was approximately 30 mm. The magnifying endoscopy with NBI (M-NBI) showed that vessels of the lesion with an epithelial circle (VEC) pattern. An irregular microvascular and microsurface architecture was observed in the circular intervening part between crypts lined by circular marginal crypt epithelium. The microvascular architecture showed dilated and tortuous atypical vessels with differences in the caliber (**Figures 1C, D**). Besides this, the VEC pattern shown by M-NBI is a promising marker for preoperative diagnosis of papillary adenocarcinoma (5). Based on the details above, we predicted that it is a lesion with a diameter of 30 mm and an infiltration depth of T1a (M). Therefore, an endoscopic submucosal dissection was performed.

**Figure 2:**
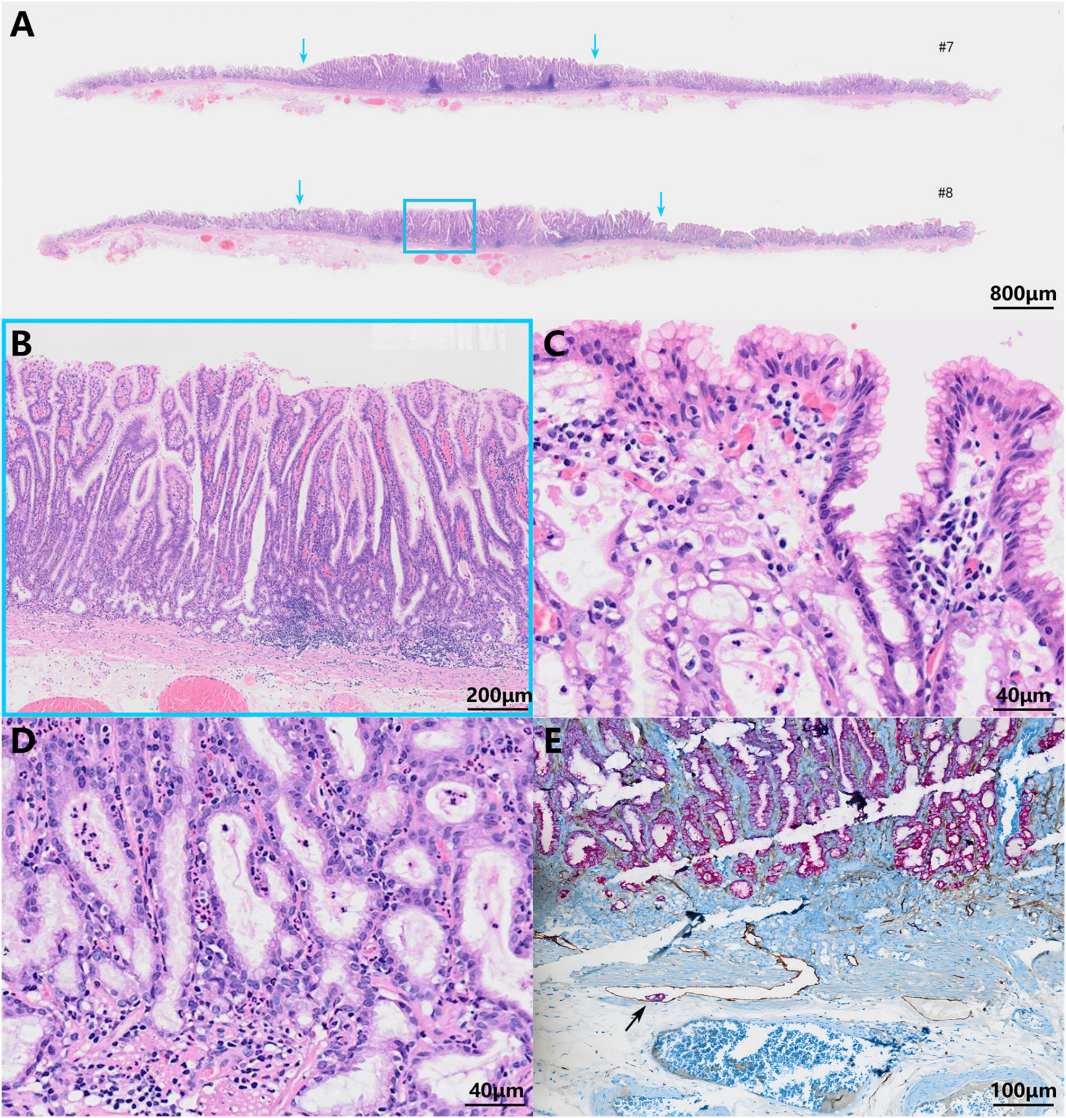
Pathohistological images of the resected specimen. Low-power field **(A)** and high-power field **(B–D)** images of hematoxylin and eosin-stained specimens. The scale bars represent 800 μm **(A)**, 200 μm **(B)**, 40 μm **(C, D)**, and 100 μm **(E)**. The blue arrows show the boundaries of the tumor **(A)**. The blue frame indicates the lesion’s superficial dense tubular and papillary structures and deeper pyloric gland-like glandular ducts **(B)**. The tumor cells were similar to foveolar epithelium **(C)** and pyloric gland cells **(D)** with low-grade cell atypia and obvious architectural atypia. A lymphovascular invasion was visible (indicated by the black arrow) **(E)**.

**Figure 3:**
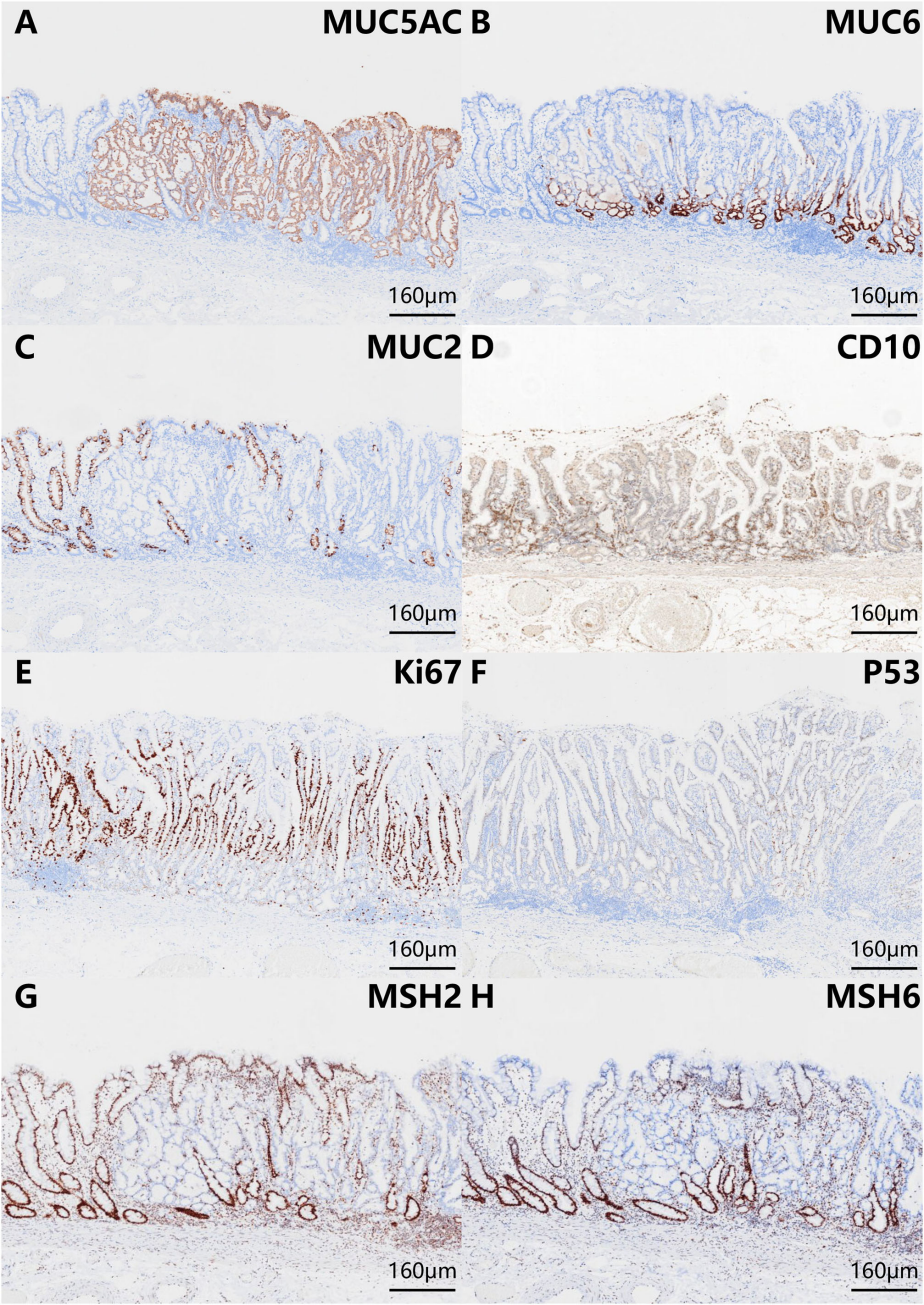
Phenotypic marker expression by immunohistochemistry staining with MUC5AC **(A)**, MUC6 **(B)**, MUC2 **(C)**, CD10 **(D)**, Ki-67 **(E)**, P53 **(F)**, MSH2 **(G)**, and MSH6 **(H)**. The scale bars represent 160 μm. The superficial foveolar-type papillary adenocarcinoma was positive for MUC5AC. The deep pyloric gland-type tubular adenocarcinoma was positive for MUC6. Both layers (the superficial part and the deep part) were negative for MUC2 and CD10. The expression of Ki-67 was high in the superficial foveolar-type papillary adenocarcinoma but low in the deep pyloric gland-type tubular adenocarcinoma. P53 was wild-type expression. The expression of MSH2 and MSH6 protein was lost.

**Figure 4:**
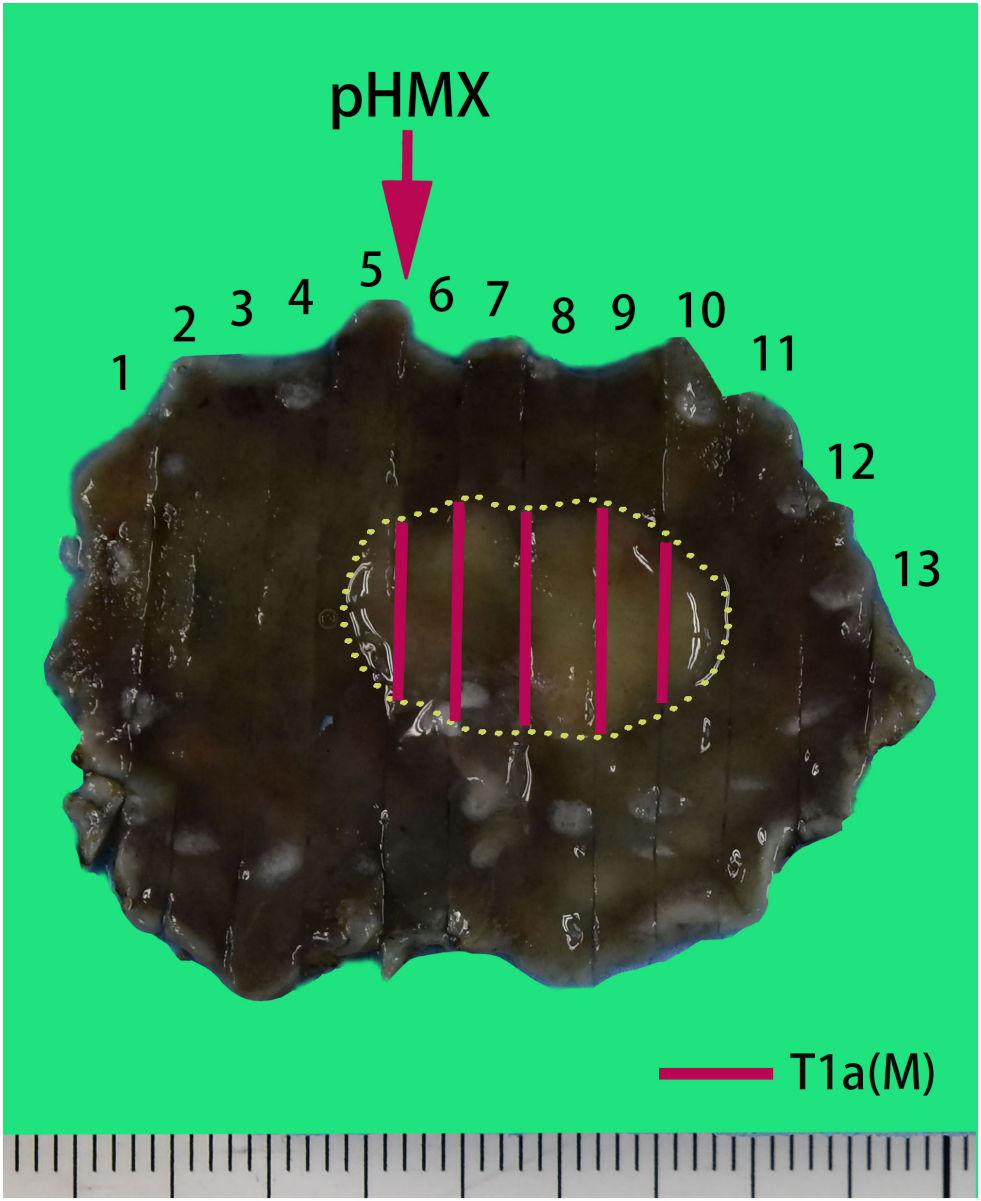
Pathological recovery diagrams. Very well-differentiated gastric adenocarcinoma of gastric type, pT1a (M), Ly1, V0, pHM0, and pVM0. The specimen was 44 mm × 32 mm and type 0-IIa, and the cancerous lesion was 18 mm × 16 mm with a red marking in the yellow circle.

The histopathologic examination of the resected specimen revealed that the surrounding mucosa of the elevated lesion was atrophic gastric fundic glands with intestinal metaplasia (**Figure 2A**). The lesion was located in the mucosal layer with a LVI (**Figure 2E**). The horizontal resection margin and the vertical margin were negative, the lesion’s superficial layer was a dense tubular and papillary structure resembling the foveolar epithelium but without a differentiation gradient, and some of the tubular and papillary structures were elongated and extended to the whole mucosa. Moreover, the deeper layer of the lesion was pyloric gland-like glandular duct (**Figure 2B**). In the magnified images, the superficial part of the elevated lesion had obvious architectural atypia and low-grade cellular atypia. The nuclei of the lesion were round to oval with disturbed polarity, and the cell cytoplasm was translucent and rich in mucus but lack mucus stratification (**Figure 2C**). These cells were MUC5AC positive (**Figure 3A**). Furthermore, the deep pyloric gland-like cells, which were positively stained for MUC6 (**Figure 3B**), proliferated and differentiated to form irregular, fused glandular ducts and eventually led to the pyloric gland-type tubular adenocarcinoma (**Figure 2D**). Both layers, namely, the superficial part and the deep part, displayed negativity for MUC2 (**Figure 3C**) and CD10 (**Figure 3D**), which was consistent with gastric type adenocarcinoma. The Ki-67 labeling index was high in the superficial foveolar-type papillary adenocarcinoma but low in the deep pyloric gland-type tubular adenocarcinoma (**Figure 3E**), and P53 was a wild-type expression (**Figure 3F**) in the lesion. The expression of MSH2 (**Figure 3G**) and MSH6 (**Figure 3H**) protein was lost. Therefore, the final pathological diagnosis was a type 0-IIa, 18 mm × 16 mm, VWDA of gastric type, pT1a, Ly1, V0, pHM0, and pVM0 (**Figure 4**).

## Discussion

VWDA is a rare type of gastric adenocarcinoma, which accounts for just 0.2%–1.9% of all primary gastric cancers (6). It is a lesion with low cell proliferation capacity in the early stage and low submucosal infiltration capacity (2). In our case, the Ki-67 positive index of the deeper pyloric gland-type tubular adenocarcinomas was low (5%). In addition, the lack of P53 mutations serves as additional evidence for its inert behavior (2, 7, 8). Similar findings have been reported in the literature—for example, the low Ki-67 positivity index (13%) of cancer infiltrating the submucosal layer suggests the slow growth and low invasiveness of VWDA (2, 9).

However, the higher Ki-67 positive index (70%) for the upper foveolar-type papillary adenocarcinoma indicated the higher malignant degree of gastric cancer. It has been reported that most papillary gastric adenocarcinomas (PGA) present with a gastric phenotype and are prone to lymphovascular invasion and lymph node metastasis (LNM) (10). Although PGA has been classified into well-differentiated gastric adenocarcinoma, a subtype known for its low malignant potential (11), the biological behavior of PGA is characterized as having a high malignant potential, a higher positive rate of liver metastasis (12) and LNM (11), and poorer surgical outcome (12) compared with non-PGA carcinomas. Especially when the cancer invades the submucosa or deeper, PGA of gastric or gastric-dominant type present with 100% LVI and 56% LNM rate (13). Furthermore, the loss of mismatch repair protein expression (MSH2 and MSH6) in this case indicates that it has MSI-H. The study of Arai Tet et al. (14) also showed that PGAs have a higher proportion of MSI-H (42%), especially in early-stage cancer. In addition, Uesugi et al. (15) showed that tubular adenocarcinomas with papillary structures showed a higher proportion of MSI than simple tubular adenocarcinomas. Thus, the presence of papillary structures is a key morphological characteristic that may indicate MSI-H. Kim DG et al. (16) reported that MSI-H gastric cancer was related to more aggressive tumors that exhibited deeper local invasion as well as LVI, with a trend toward an increase in LNM. This is consistent with the biological characteristics of LNM in papillary VWDA, even in intramucosal papillary VWDA (13). Therefore, the superficial dense tubular and papillary structures in the present case may be related to the LVI, which suggests that the VWDA of our case has similar biological behavior and prognosis as the early PGAs.

Foveolar-type (gastric type) adenocarcinomas possess foveolar gland structures in which stem cells, thought to be present in the proliferative region of the neck of the glands, can differentiate into surface mucous epithelial and glandular cells (17). During the process, foveolar-type (gastric type) adenocarcinomas could differentiate into kinds of gastric or intestinal adenocarcinomas, and the malignant potential is gradually increased. In this case, despite the low proliferation index of Ki-67, the obvious cribriform and reticulum-like structure of the deep pyloric adenocarcinoma is a sign of intermediate differentiation, that is to say, if other gastric adenocarcinoma components are present, the biological behavior and prognosis of foveolar-type adenocarcinomas may still predominate.

In conclusion, although this case is intramucosal adenocarcinoma, its biological behavioral characteristics are consistent with those of early PGAs reported, indicating that the presence of foveolar-type papillary adenocarcinoma component might indicate a higher degree of malignancy of the tumor. However, the specific regulatory mechanisms need to be further elucidated through more case studies. Most importantly, we recommend a close follow-up of patients with this characteristic.

## Conclusion

We report a rare case of VWDA of gastric type with LVI. It suggests that the foveolar-type papillary adenocarcinoma components may be one of the factors affecting the prognosis and biological behavior of the tumor. In the future, more cases should be accumulated to further investigate the correlation between the papillary components and the biological behavior of VWDAs. Moreover, the specific regulatory mechanisms need to be further elucidated.

## Data availability statement

The original contributions presented in the study are included in the article/Supplementary Material. Further inquiries can be directed to the corresponding authors.

## Ethics statement

The studies involving humans were approved by The Second Hospital of Shandong University. The studies were conducted in accordance with the local legislation and institutional requirements. The human samples used in this study were acquired from a by- product of routine care or industry. Written informed consent for participation was not required from the participants or the participants’ legal guardians/next of kin in accordance with the national legislation and institutional requirements. Written informed consent was obtained from the individual(s) for the publication of any potentially identifiable images or data included in this article.

## Author contributions

JC: Writing – review & editing, Writing – original draft. XC: Writing – review & editing, Funding acquisition. HW: Writing – review & editing, Supervision. CZ: Writing – review & editing, Conceptualization.
